# Bat SARS-Like WIV1 coronavirus uses the ACE2 of multiple animal species as receptor and evades IFITM3 restriction *via* TMPRSS2 activation of membrane fusion

**DOI:** 10.1080/22221751.2020.1787797

**Published:** 2020-07-09

**Authors:** Mei Zheng, Xuesen Zhao, Shuangli Zheng, Danying Chen, Pengcheng Du, Xinglin Li, Dong Jiang, Ju-Tao Guo, Hui Zeng, Hanxin Lin

**Affiliations:** a Institute of Infectious Disease, Beijing Ditan Hospital, Capital Medical University, Beijing, People’s Republic of China; b Beijing Key Laboratory of Emerging Infectious Disease, Beijing, People’s Republic of China; c Baruch S. Blumberg Institute, Hepatitis B Foundation, Doylestown, PA, USA; d Department of Pathology and Laboratory Medicine, Western University, London, Canada

**Keywords:** SARS-like coronavirus WIV1, ACE2 receptor, viral entry, IFITM, TMPRSS2

## Abstract

Diverse SARS-like coronaviruses (SL-CoVs) have been identified from bats and other animal species. Like SARS-CoV, some bat SL-CoVs, such as WIV1, also use angiotensin converting enzyme 2 (ACE2) from human and bat as entry receptor. However, whether these viruses can also use the ACE2 of other animal species as their receptor remains to be determined. We report herein that WIV1 has a broader tropism to ACE2 orthologs than SARS-CoV isolate Tor2. Among the 9 ACE2 orthologs examined, human ACE2 exhibited the highest efficiency to mediate the infection of WIV1 pseudotyped virus. Our findings thus imply that WIV1 has the potential to infect a wide range of wild animals and may directly jump to humans. We also showed that cell entry of WIV1 could be restricted by interferon-induced transmembrane proteins (IFITMs). However, WIV1 could exploit the airway protease TMPRSS2 to partially evade the IFITM3 restriction. Interestingly, we also found that amphotericin B could enhance the infectious entry of SARS-CoVs and SL-CoVs by evading IFITM3-mediated restriction. Collectively, our findings further underscore the risk of exposure to animal SL-CoVs and highlight the vulnerability of patients who take amphotericin B to infection by SL-CoVs, including the most recently emerging (SARS-CoV-2).

## Introduction

The outbreak of severe acute respiratory syndrome (SARS) during 2002–2003 caused 774 deaths in 8096 infected people in the world [1]. Although this outbreak was relatively small in scale, it posed significant challenge in medical community and had a deep socioeconomic impact. Where the SARS-CoV was originated and how it entered into human population are two questions of great scientific and practical importance and have been extensively investigated ever since. It is now generally believed that the direct progenitor of SARS-CoV was generated by sequential recombination events among bat SARS-like CoVs (SL-CoVs) and used civet cat as intermediate host to jump to humans [2,3]. Evidences supporting this bat origin and interspecies jumping theory include: (*i*) genetically diverse SL-CoVs, sharing 78%–96% of genome sequence identity with SARS-CoV, were identified in Chinese horseshoe bat (*Rhinolophus sinicus*, bat-Rs) in China [4–11]; (*ii*) civet isolates share 99.6% nucleotide sequence identity with human isolates, suggesting a very recent cross-species transmission [12–15]; and (*iii*) no sign of overt diseases were observed in horseshoe bats carrying SL-CoVs. The fact that bats are the nature reservoir of SARS-CoV [3] and the recently emergence of SARS-CoV-2, that is also believed to originate from bat SL-CoV [16,17] and be introduced into humans with or without an intermediate host, strongly demonstrate that the SL-CoVs from bats and other species present a continuous threat to human health.

Cell entry is the first step of virus life cycle and is the key determinant for host range and tissue tropism of viruses. It can be divided into two steps: cell attachment and membrane fusion. For coronaviruses, the former step is primarily mediated by the specific binding of envelope spike (S) protein to their cellular receptors. Following receptor binding, the S protein undergoes conformational changes, either triggered by receptor binding and/or low pH in order to overcome the energy barrier for membrane fusion. The efficiency of cell entry is affected by many factors, including specific receptor usage, binding to attachment factors, susceptibility of S protein to protease cleavage, and/or acid-induced conformation changes [18,19]. Cell entry of SARS-CoV has been well characterized [19,20]. For example, human angiotensin converting enzyme 2 (ACE2) is the *bona fide* cellular receptor for SARS-CoV [21]. SARS-CoV can also use some other animal ACE2 orthologs as receptor. These include civet cat, raccoon dog, rhesus monkey, ferret, mink, and feline [22–26], but not ACE2 of horseshoe bat (*Rhinolopphus. pearsonii*) and rat [27,28]. The activity of these ACE2 orthologs to mediate SARS-CoV cell entry is consistent with the susceptibility of these animals to SARS-CoV infection [12,29–31]. The S protein of SARS does not have furin cleavage sites and is thus not cleaved during biogenesis in producing cells, but it can be cleaved during infection of target cells by multiple cellular proteases, such as endosomal cathepsin L, type II transmembrane serine proteases (TTSPs), human airway trypsin-like protease (HAT), or transmembrane protease, serine 2 (TMPRSS2), to facilitate efficient entry [32–35]. Other proteases, such as trypsin, thermolysin, elastase and factor Xa can also cleave SARS S protein, and the cleavage enhances infectivity as well [32,33,36]. On the contrary, little is known about the cell entry process of bat SL-CoVs. A group of genetically diverse SL-CoVs were identified from multiple species of horseshoe bats in China and Europe [4,5,37], but none of them could use ACE2 as entry receptor until the identification of two bat SL-CoV isolates, WIV1 and WIV16, in bat-Rs [9–11,38]. These two isolates are able to use ACE2 molecules from bat-Rs, human, and civet for cell entry, implying that they have the potential to probably jump from bat to human without an intermediate host. These finding support an early hypothesis that the progenitor of SARS-CoV was probably jumped from bat to human, and used civet cat as a secondary host and reservoir for continued infection [39]. However, those studies were all done by using live viruses and the entry step of these bat SL-CoVs has not been explicitly characterized. In addition, whether or not ACE2 orthologs from other wild animals can be used as receptor for these bat SL-CoVs remains to be determined.

IFITMs are the primary host defense factors that restrict the entry of multiple enveloped viruses and play a critical role in viral pathogenesis [40,41]. Interestingly, despite the conserved structural architecture of spike proteins of human coronaviruses [42], IFITMs efficiently restrict the endosomal entry of all the human coronaviruses, expect for HCoV-OC43, which hijacks IFITMs as its entry factors to promote its infection [43–45]. Not surprisingly, many CoVs have evolved strategies to circumvent IFITM-mediated endosomal restriction by utilizing multiple proteases to activate spike protein at or close to plasma membrane [43,46]. Thus far, no study has been carried out to examine the restricting effect of IFITMs on SL-CoVs entry and capability of these viruses to evade IFITM restriction.

In this study, we examined the cell entry process of a bat SL-CoV isolate WIV1. Our results showed that multiple wild animal ACE2 orthologs were able to support WIV1 pseudotype virus entry. While the entry of WIV1 and other SL-CoVs, including civet isolates B039G and A022G, was inhibited by IFITM proteins, WIV1 could use trypsin-like protease and TMPRSS2 to bypass IFITM-mediated restriction. We also found that amphotericin B, an antibiotic used for treatment of patients with severe fungal infections and leishmaniasis, significantly promoted SL-CoV spike-driven infection by disrupting IFITM3-mediated restriction. Our study reported herein has important implications on the potential intermediate wild hosts of bat SL-CoV WIV1 and risk of patients who take amphotericin B treatment for infection of SL-CoVs, including the currently epidemic SARS-CoV-2.

## Materials and methods

### Cell lines and reagents

293 T cells and Lenti-X 293 T cells were cultured in Dulbecco's modified Eagle's medium (DMEM; Gibco). FLP-IN T Rex293 cell lines (Invitrogen) and its derived cell lines expressing individual IFITM proteins were cultured in DMEM supplemented with zeocin (100 μg/ml) and blasticidin (10 μg/ml) or 5 μg/ml blasticidin and 250 μg/ml hygromycin, respectively [47]. All growth medium was supplemented with 10% fetal bovine serum (FBS), 110 mg/L sodium pyruvate, and 4.5 g/L D-glucose.

### Construction of ACE2 plasmids

ACE2 molecules of human (*Homo sapiens*), civet (*Paguma larvata*), and rat (*Rattus norvegicus*) were cloned into a modified pcDNA3.1-cmyc/C9 vector (Invitrogen) and previously described [27,48]. ACE2 protein expressed from this vector has a c-myc tag at the N-terminus and a C9 tag at the C-terminus. An Age I site was engineered right downstream of the signal peptide sequence (nt. 1-54) of hACE2. The ACE2 molecules of Chinese ferret badger (*Melogale moschata*), raccoon dog (*Nyctereutes procyonoides*), Mexican free-tailed bat (*Tadarida brasiliensis*), hog badger (*Arctonyx collaris*), and domestic cat (*Felis catus*) were described previously (Hanxin Lin, Ph. D Thesis Dissertation. “Molecular interaction between the spike protein of human coronavirus NL63 and ACE2 receptor” McMaster University, Health Science Library. https://discovery.mcmaster.ca/iii/encore/record/C__Rb2023203__SMolecular%20interaction%20between%20the%20spike%20protein%20of%20human%20coronavirus%20NL63%20and%20ACE2%20receptor%20Lw%3D%3D%20by%20Hanxin%20Lin__Orightresult__U__X4?lang=eng&suite=def). Briefly, ACE2 cDNA was amplified using BD SMART™ RACE cDNA Amplification Kit (BD Biosciences Clontech). The total RNAs of these animals, except bat, were extracted from the mixture of lung and kidney tissues using RNeasy Mini kit (QIAGEN, ON). The total RNAs of bat were extracted from Tb-1 Lu cell culture. Two cDNA populations, 5′-RACE-Ready cDNAs and 3′-RACE-Ready cDNAs, were synthesized from the total RNAs according to the manufacturer's instruction. Overlapping DNA fragments that cover the full-length ACE2 genes were amplified by nested PCR with *Pfx* DNA polymerase (Invitrogen) with two primer pairs: internal forward primer GSP1 5′-CCCTTTGGACAGAAACCAAACATAGATGT-3′ (nt. 850-878 of multiple aligned ACE2s) and external backward primer 5′-CTAAAATGAAGTCTGAACATCATCATC-3′ (nt. 2395-2418) or universal primer A mix (UPM) that is supplied in the kit; internal backward primer GSP2: 5′- CCRACKATVTYYCGCTTCATCTCCCACCA-3′(nt. 1429-1458) and external forward primer 5′-ATGTYVRGYTCHTBCTGGCTCCTTCTCAG-3′ (nt. 1-29). The PCR fragments were cloned into pGEM-T vector (Promega, Madison, WI). Three clones of each fragment were sequenced. Based on the determined sequences of specific animal species, forward primers with an Age I site and backward primers with a Kpn I site were designed. These primers were then used to amplify the full-length ACE2s using the overlapping PCR strategy recommended by the manual of BD SMART™ RACE cDNA Amplification Kit. The full-length PCR products of ACE2 molecules were cloned into Age I/Kpn I-digested pcDNA3.1-cmyc/C9 vector vector, and subject to sequencing to confirm their correctness. The nucleotide sequence of Chinese horseshoe bat (*Rhinolophus sinicus*, Rs) ACE2 obtained from NCBI database (KC881004.1) was synthesized and cloned into pcDNA3.1-cmyc/C9 vector with the same strategy.

### Construction of plasmids expressing S and RBD proteins of SL-CoVs

The nucleotide sequence of WIV1 S protein was obtained from NCBI database (AGZ48828.1). According the method described by Gregory J. Babcock et al. [49], the codon-optimized WIV1 S gene was synthesized based on the sequence of WIV1 S, and then cloned into pSecTag2 vector. The plasmids encoding full-length codon-optimized S protein of Tor2, GD03, SZ3, B039G and A022G were synthesized with the same strategy. The S genes were also cloned into a modified pSecTag2 vector (N-myc-pSecTag2). The S protein expressed from this vector has a myc tag at the N-terminus. To produce soluble RBD-Ig fusion proteins, the receptor-binding domain (RBD) of these SL-CoVs were cloned into a soluble protein expression vector, pSecTag2/Hygro-Ig vector, which contains human IgG Fc fragment and mouse Ig k-chain leader sequence [50].

### Western blot assay

As previously described, the expression of ACE2 proteins or RBD-Ig fusion proteins were examined by western blot [50]. Briefly, lysates or culture supernatants of 293 T cells transfected with plasmid encoding ACE2 orthologs and RBD-Ig of SL-CoVs were collected, boiled for 10 min, and then resolved by 4∼12% SDS-PAGE. To detect the incorporation of N-myc tagged S protein into pseudotyped particles, 8 mL of the respective pseudotypes were loaded onto a 20% (w/v) sucrose cushion and underwent centrifugation (25.000 g for 150 min at 4_°C). Then, the supernatant was removed and the pellet was mixed with 4×SDS-sample buffer, boiled for 10 min and resolved by 8% SDS-PAGE. A PVDF membrane containing the proteins transferred from SDS-PAGE was blocked with blocking buffer (5% nonfat dry milk in TBS) for 1 h at room temperature and probed with primary antibody overnight at 4°C. The blot was washed three times with washing buffer (0.05% tween-20 in TBS), followed by incubation with secondary antibody for 1 h at room temperature. After three time washes, the proteins bounded with antibodies were imaged with the Li-Cor Odyssey system (Li-Cor Biotechnology).

### Production of pseudotyped virus

Following the standard protocol of calcium phosphate transfection, Lenti-X cells in 10 cm plate were co-transfected by 20μg of HIV-luc and 10 μg of CoVs spike gene plasmid. At 48 h post-transfection, 15 ml supernatant was collected and passed through a 0.45-µm pore size filter to purify the virus. The virus was stored at −80°C.

### Virus entry assay

Each well of 293 cells in a 96-well plate was transfected with 0.1 µg of ACE2 plasmid DNA by using Lipofectamine 2000 (Invitrogen). At 48 h post-transfection, the medium was removed, then 100 µl of p24-normalized (10 ng) of pseudotype virus of SARS and SL-CoV WIV1 was added into each well and incubated at 37°C for 2 h. The virus was then removed, and 100 µl of fresh medium was added into each well for further incubation. Two days later, the medium was removed and the cells were lysed with 30 µl 1× lysis buffer (Promega), incubated at room temperature for 15 min. For measurement of luciferase (Luc) activity, cell lysate was mixed with 50 µl luciferase substrate (Promega) and Luc activity was measured using a luminometer (Top-Counter instrument, PerkinElmer). For each ACE2, four wells were tested in a single experiment, and at least three repeat experiments were carried out. The Luc values were normalized to human ACE2 and were used for plotting.

To test Amphotericin B (AmphoB) effect on viral entry, ACE2-transfected 293 cells in a 96-well plate was mock-treated or treated with 1μM AmphB for 1 h and followed by infection with respective pseudoviral particles. After 2 h inoculation, the virus was then removed, and 100 µl of fresh medium was replenished into each well for further incubation. Luciferase activity was measured at 48 h post-infection.

### Syncytial formation assay

293 T cells, approximately 70∼90% confluent on 12-well plate, were transfected with 1.6 μg plasmid encoding SARS-CoV (Tor2 strain) S protein or ACE2 of different animals (human, bat, rat, hog bagger, ferret bagger, raccoon dog, and civet cat). At 24 h post-transfection, 293 T cells expressing SARS-CoV spike protein were mixed at a 1:1 ratio with 293 T cells expressing ACE2 and plated on 12-well plate. Multinucleated syncytia were observed 24 h after the cells were mixed as described previously [51].

To quantitatively measure syncytium formation, 293 T cells seeded in 6-well plates were transfected with plasmids encoding ACE2 orthologs or S and pCMV/T7 pol or pT7/Luc by using Lipofectamine 2000 (Invitrogen). At 24 h post-transfection, 293 T cells were trypsinized and mixed in equal amounts between the two sets of transfected cells. The mixed cells were seeded into 96-well plates at a density of 5 × 10^4^ cells per well. After another 24 h of incubation at 37°C, cells were lysed, followed by adding luciferase substrate (Promega) to measure the firefly luciferase activities with a luminometer (Top-Counter instrument, PerkinElmer) [51].

### Effect of TMPRSS2 on cellular entry of WIV1

T Rex 293 T cells in six-well plates were cotransfected with a hACE2 plasmid and a pCAGGS-TMPRSS2 plasmid, or empty pCAGGS plasmid as a negative control. At 24 hr. post-transfection, T Rex 293T-hACE2 and T Rex 293T-hACE2-TMPRSS2 cells were seeded in 96-wells plates respectively. One day later, the cells were infected by WIV1 S pseudotyped lentivirus. At 48 h post-infection, luciferase activities were measured by a luminometer.

### Trypsin activity assay

ACE2-transfected FLIP-IN T Rex293-IFITM3 cells were cultured in the presence or absence of tetracycline (1μg/ml) for 24 h, and then incubated with pseudotyped particles of Tor2 (Tor2pp) and WIV1 (WIV1pp) pseudotyped virus, centrifuging at 4 °C for 35 min at 3500×g. Transduced cells were treated with 5 μg/ml TPCK-trypsin or DMEM without FBS at 37°C for 13 min. Then, DMEM including 10% FBS were added to culture for 48 h. Luciferase activities were measured at 48 h post-infection.

## Results

### Multiple ACE2 orthologs serve as receptor for bat SL-CoV WIV1

**Figure 1.**
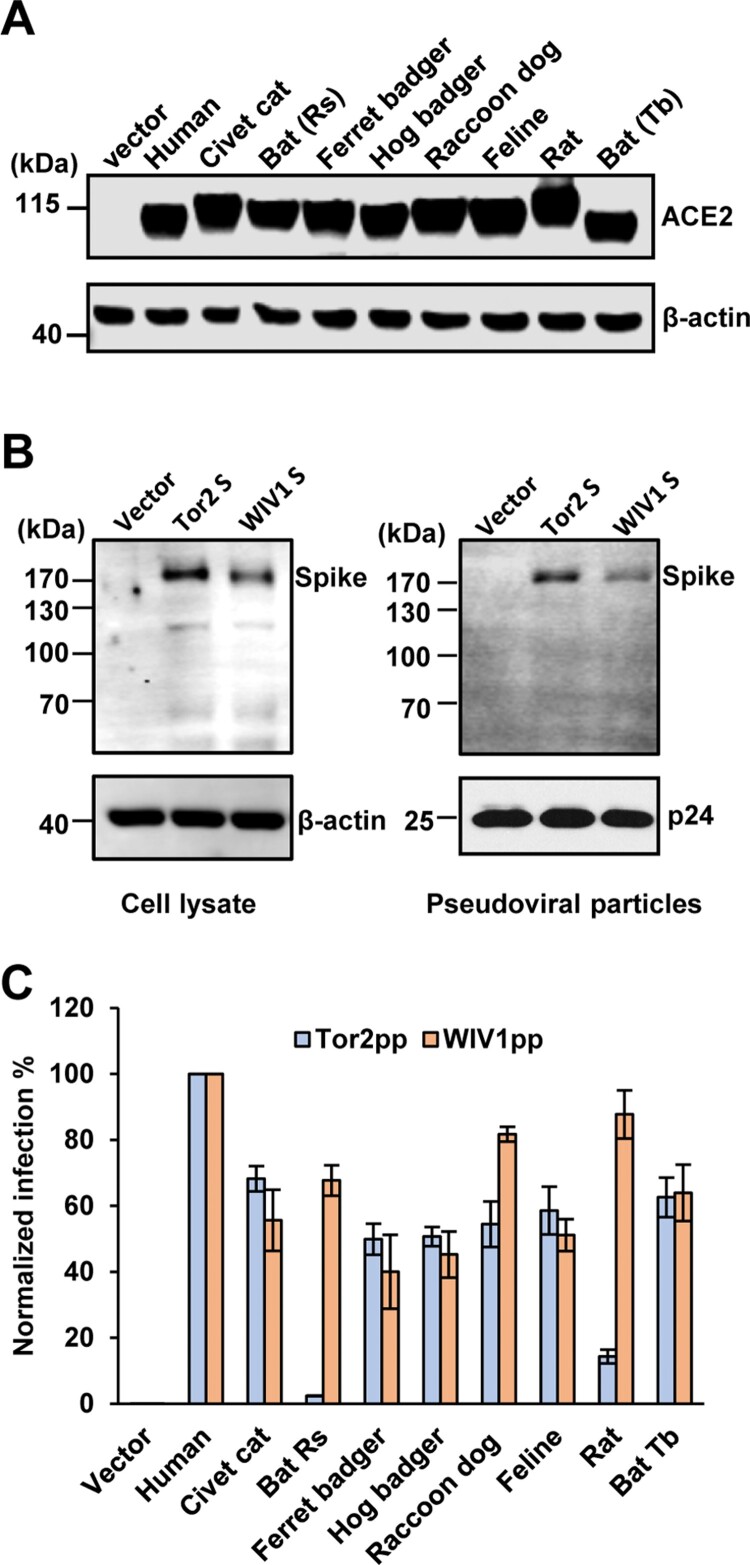
Multiple ACE2 orthologues supported spike protein-mediated entry and membrane fusion of SARS-CoV and bat SL-CoV WIV1. (A) Transient expression of ACE2 orthologs in 293 T cells. The cell lysates were detected by western blot assay, using an anti-C9 monoclonal antibody. (B) Detection of WIV1 and Tor2 S expression (left panel) and pseudotype incorporation (right panel) by western blot using an antibody targeting the N-terminal myc tag added to the viral S proteins. β-Actin (cell lysates) and p24 (pseudoviral particles) served as loading controls. (C) HIV-1-Luc-based pseudotyped virus entry. 293 T cells were transfected with empty vector pcDNA3.1 or ACE2s orthologs. At 48 h. post-transfection, the cells were infected by pseudotyped virus particles of SARS-CoV Tor2 (Tor2pp) or WIV1 (WIV1pp). At 48 h. post-infection, luciferase signal of each set of Tor2pp or WIV1pp infection was measured and normalized to human ACE2, respectively. Error bars reveal the standard deviation of the means from four repeats.

SL-CoV WIV1 was isolated from Chinese horseshoe bat (Rhinolophus sinicus, bat-Rs) and known to use ACE2 from bat-Rs, civet cat and human as receptor to enter host cells [9]. However, whether the ACE2 molecules of other animal species can serve as receptor for WIV1 remains unknown. To address this question, we cloned the ACE2 complementary DNA (cDNA) from ferret badger, hog badger, raccoon dog, feline, and Mexican free-tailed bat (bat-Tb). The ACE2 molecules from these animals as well as bat-Rs, civet cat, human and rat were transiently expressed in 293 cells (Figure 1(A)). The cells were then infected by HIV-1 pseudotyped particles with the spike protein derived from WIV1 (WIV1pp) or SARS-CoV severe isolate Tor2 (Tor2pp). The expression of S protein in package cells and incorporation in pseudotyped particles was shown in Figure 1(B). The receptor activity was measured and normalized to human ACE2 (Figure 1(C)). The results showed that, for Tor2pp, human ACE2 was the most efficient receptor. ACE2 orthologs of civet cat, ferret badger, hog badger, raccoon dog, feline and bat-Tb (a Mexican free-tailed bat) could supported Tor2pp entry at a level of 50%–68% of hACE2, while ACE2 orthologs of bat-Rs and rat were inefficient receptor (2% and 14% of hACE2, respectively). Similar to Tor2pp, human ACE2 was also the most efficient receptor for WIV1pp. However, all the other animal ACE2 orthologs, including bat-Rs and rat, could efficiently support WIV1pp entry at a level of 40%–88% of hACE2.

**Figure 2.**
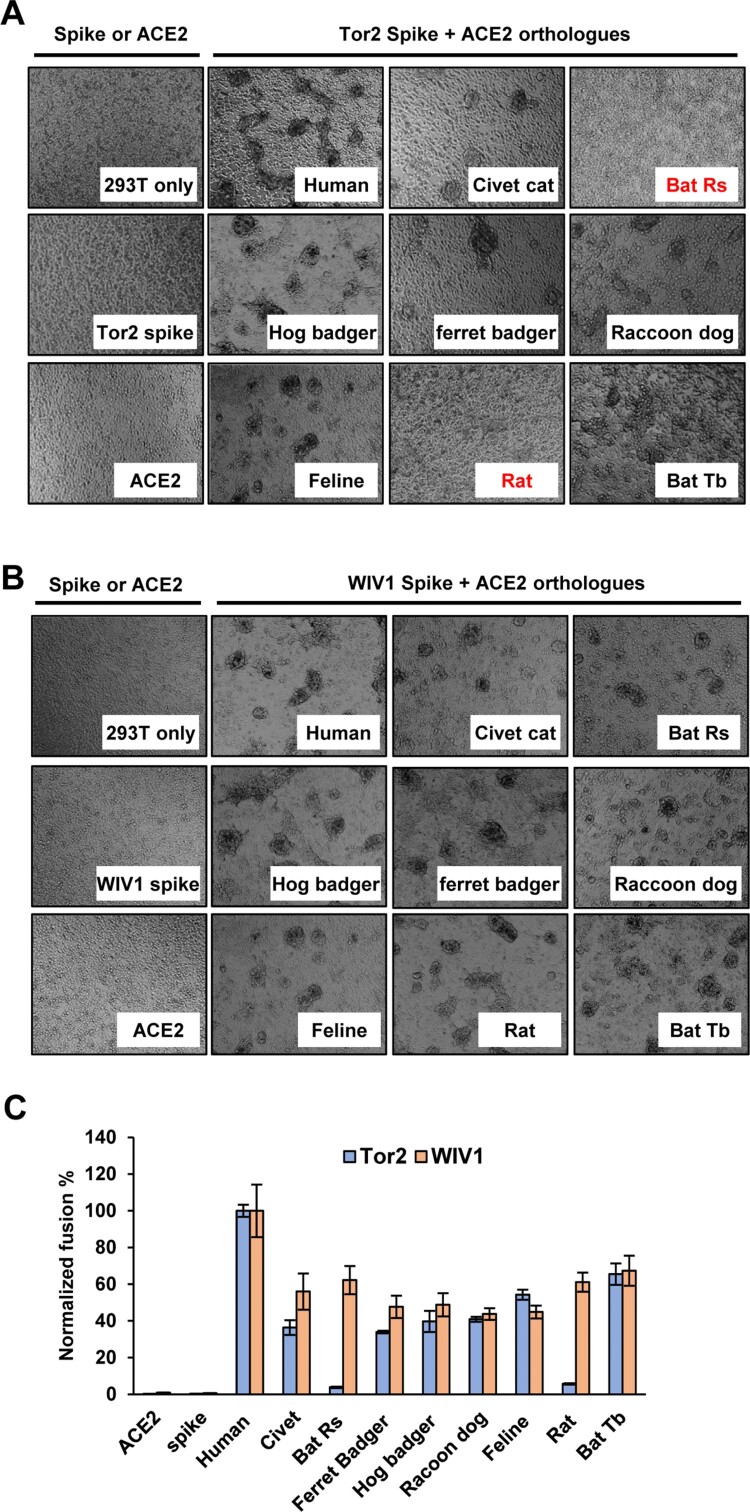
Multiple ACE2 orthologues supported WIV1 spike protein-mediated membrane fusion. (A) and (B) Syncytia formation assay. 293 T cells transfected with plasmid DNA of S gene of Tor2 (A) or WIV1 (B) were mixed at a 1:1 ratio with those cells transfected with plasmids encoding different animals ACE2s. Twenty-four hours later, syncytia formation was observed. (C) 293 T cells cotransfected with plasmids encoding either ACE2 orthologs and T7 polymerase or spike protein and T7/luciferase. For syncytium formation, the cells were mixed at a 1:1 ratio and cultured for 24 h. Luciferase activities in the cell lysates were determined, normalized to the human ACE2 mixed with S, and then expressed as means ± standard deviations (*n* = 4).

We also performed a syncytia formation assay to assess the level of membrane fusion triggered by receptor binding. We found that 293 T cells expressing ACE2 orthologs of human, civet cat, ferret badger, hog badger, raccoon dog and bat-Tb formed large syncytia with cells expressing SARS-CoV Tor2 S protein. However, fewer syncytia were observed between 293 T cells expressing bat-Rs ACE2 or Rat ACE2 and cells expressing Tor2 S protein (Figure 2(A)). In marked contrast, cells expressing any of the ACE2 orthologs formed large syncytia with cells expressing WIV1 S protein (Figure 2(B)). These findings were also in line with the results from a quantitative fusion assay (Figure 2(C)). Therefore, the results of syncytia formation assay were consistent with the pseudotyped virus infection assay. These results clearly suggested that bat SL-CoV WIV1 has the potential to infect a wide range of wild animals and may directly jump to humans.

### Human ACE2 is not yet a highly efficient receptor for WIV1

**Figure 3.**
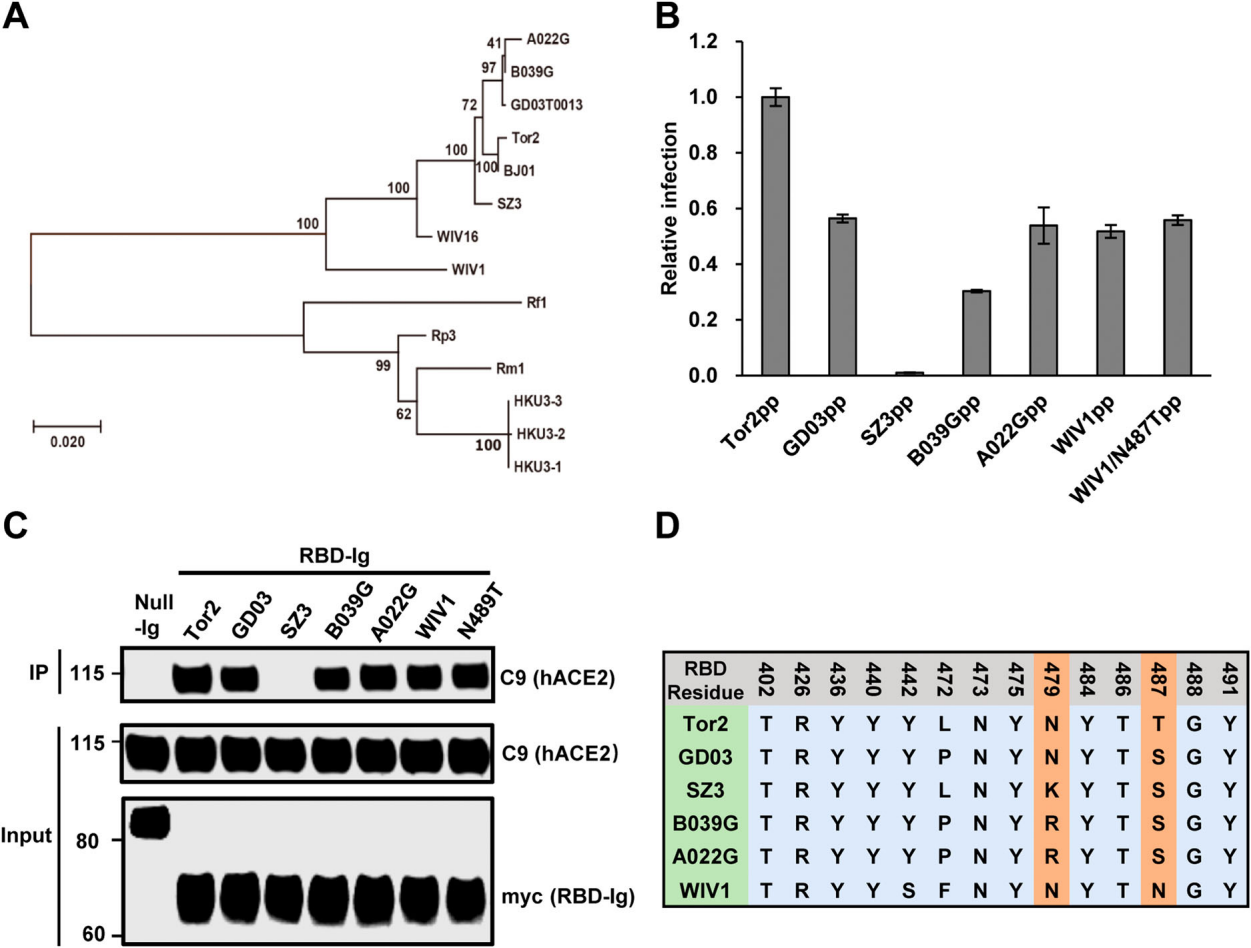
Human ACE2 is not an efficient receptor for WIV1. (A) Phylogenetic tree of SL-CoVs and SARS-CoVs based on the amino acid sequence of S protein. (B) Pseudotyped virus entry. 293 T cells transfected with plasmid DNA of human ACE2 were infected by similar amount of pseudotyped virus particles of SARS-CoVs (a severe isolate Tor2 and a mild isolate GD03), WIV1, and civet cat SL-CoVs (SZ03, B039G and A022G). At 48 h. post-infection, the luciferase activities were measured and normalized to Tor2pp. Error bars reveal the standard deviation of the means from four repeats. (C) The interactions of human ACE2 and receptor-binding domain (RBD) of S proteins of SARS-CoVs and SL-CoVs were detected by IP assay by using anti-C9 antibody and anti-myc antibody. (D). Sequence alignment of 14 amino acid residues in the RBD that are critical to human ACE2 binding.

Receptor usage of SARS-CoV is primarily determined by a so-called receptor-binding domain (RBD) in the S protein [52]. Some amino acid residues in the RBD directly interact with ACE2 and play a critical role in receptor binding, virus entry and interspecies transmission [48]. To further understand the cell entry mechanism of WIV1, we compared the RBD-hACE2 binding and pseudotyped virus entry between WIV1 and a group of SL-CoVs and SARS-CoVs. These include SARS-CoV Tor2 (a severe human isolate during 2002–2003 pandemic), SARS-CoV GD03 (a mild human isolate from sporadic infection in 2003–2004), and three civet cat SL-CoVs (SZ3, A022G and B039G). These CoVs are closely related and form a single cluster based on the phylogenetic analysis of the S gene (Figure 3(A)). By using similar amount of pseudotyped viruses to infect 293 cells stably expressing hACE2, we found that WIV1, GD03, B039G and A022G had a similar level of cell entry (55%–60% of Tor2), while human ACE2 barely supported the entry of civet isolate SZ03 (Figure 3(B)). The efficiency of the S proteins to drive the pseudovirus infection is tightly correlated with its RBD's binding activity to human ACE2 (Figure 3(C)). Comparing the sequence variation of 14 RBD residues that are known to directly interact with human ACE2 [53], we found that the SARS-CoV isolates and SL-CoVs isolates are highly conserved except for variations at 4 residues (442, 472, 479 and 487 of Tor2 S, Figure 3(D)). Noticeably, WIV1 has Asparagine at 487, while Tor2 has Threonine at this residue. Previously, it was reported that changing of S487 to T487 could significantly enhance the hACE2 to support the cell entry of civet cat isolate SZ03 [48]. However, substitution N487 with T487 did not apparently alter WIV1 S protein-mediated infection (Figure 3(B,C)). Collectively, these results indicate that although WIV1 has a potential to directly jump to humans, it has not yet fully evolved to efficiently use human ACE2 as its receptor.

### IFITM proteins restrict SL-CoV spike-mediated viral entry

**Figure 4.**
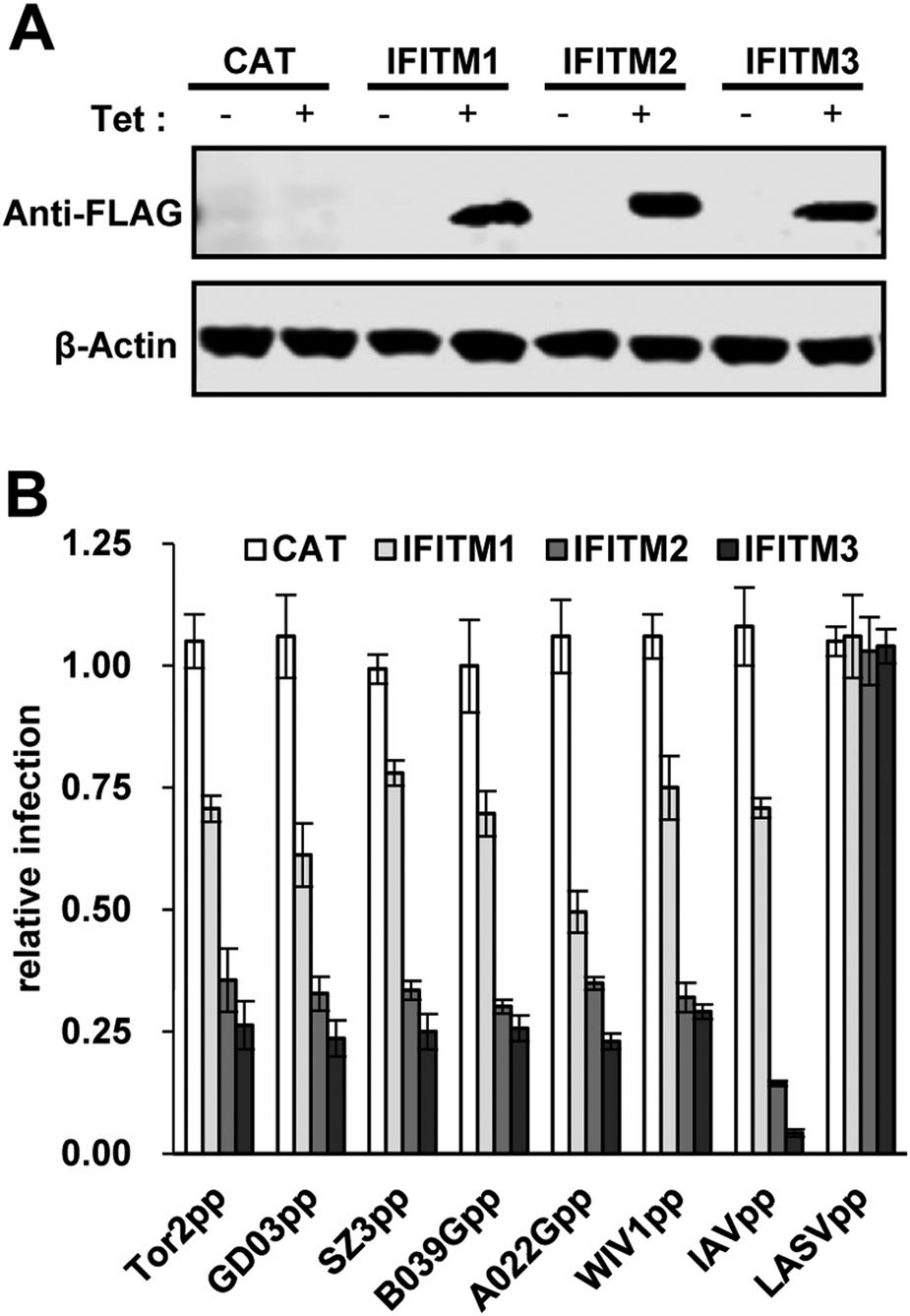
IFITM proteins restrict SL-CoV spike-mediated viral entry. (A) FLIP-IN T-Rex 293 cells which expressed CAT or indicated IFITM proteins were cultured in DMEM with/without 1μg/ml tetracycline. Twenty-four hours later, expression of IFITM proteins was tested by western blot assay by using anti-FLAG antibody. (B) ACE2-transfected FLIP-IN T-Rex 293 cells, which expressed CAT or IFITMs proteins, were cultured in the existence or of absence of tetracycline (1μg/ml) in 96-well plate, and then were infected with similar amount of pseudotyped virus particles of SARS-CoVs and SL-CoVs. Influenza virus A virus and Lassa virus (LASV) were used as positive and negative control, respectively. The luciferase activity was measured at 48 h post-infection. Relative infection activity refers to the ratio of luciferase efficiency in cells grown in 1μg/ml Tet over which in cells grown without Tet. Error bars reveal the standard deviation (n = 4).

We and others demonstrated previously that the entry of SARS-CoVs can be restricted by IFITM proteins, a group of intrinsic restriction factors impeding the entry of a broad spectrum of viruses into host cells [43,45]. To investigate if IFITMs can also inhibit the entry of SL-CoVs, we determined the efficiency of lentiviral particles pseudotyped with spike protein of WIV1 or other SL-CoVs to infect 293 cells inducibly expressing one of the three human IFITM proteins in a tetracycline-induced manner (Figure 4(A)). As expected, all three IFITM proteins did not inhibit the entry of lentiviral particles pseudotyped with the envelope protein of Lassa fever virus (LASVpp), which served as a negative control, and significantly reduced the infection of lentiviral particles pseudotyped with the envelope proteins of influenza A virus (IAV) (IAVpp), which served as a positive control (Figure 4(B)). As shown in Figure 4(B), the infection of lentiviral particles pseudotyped by the spike protein of all the SL-CoVs was efficiently inhibited by IFITMs. Of note, although the S proteins of these CoVs examined had different efficiency in using hACE2 as receptor for entry (Figure 3), their sensitivity to the restriction by a given IFITM protein is similar.

### WIV1 exploits TMPRSS2 to partially evade IFTIM3-mediated restriction

**Figure 5.**
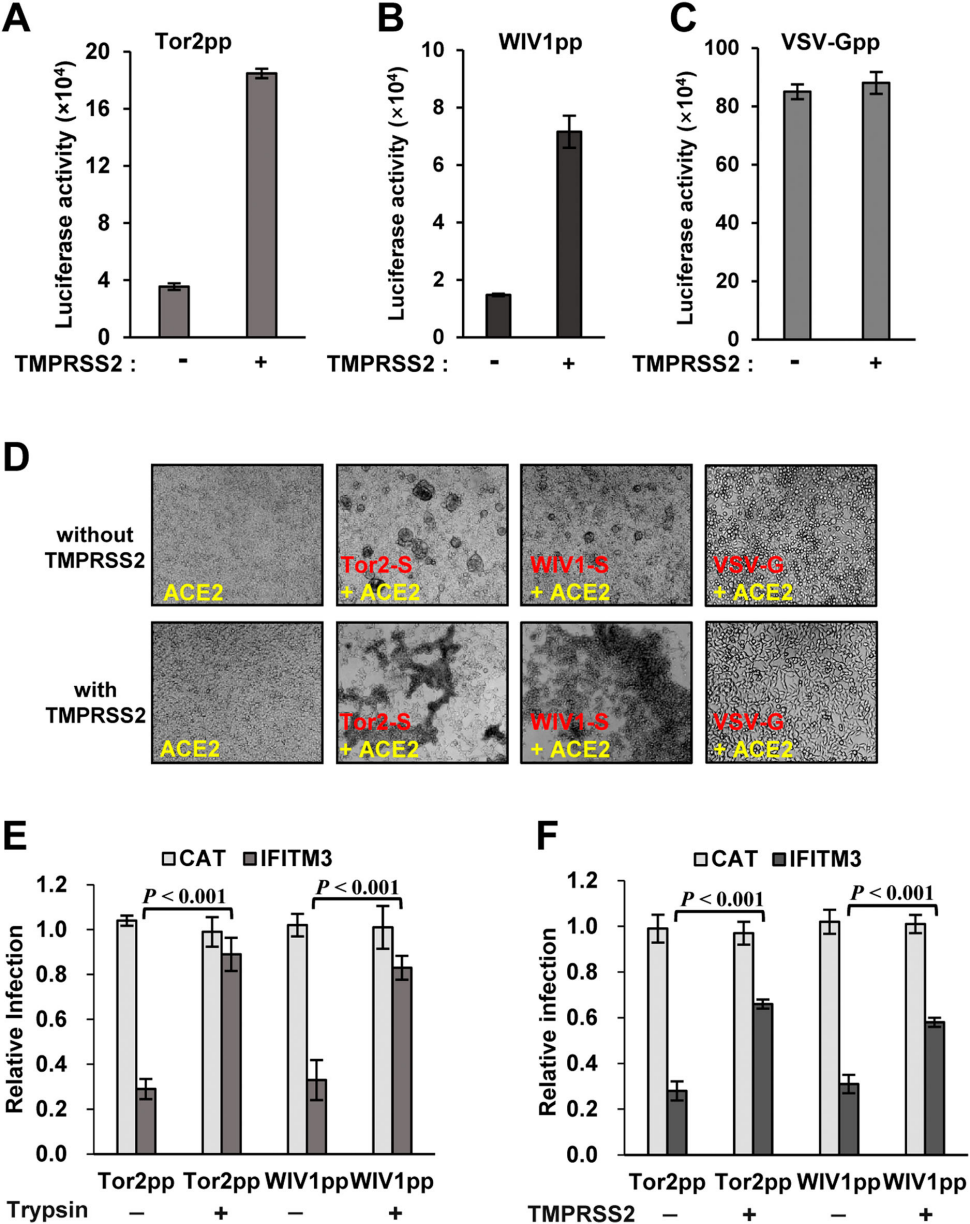
TMPRSS2 promotes WIV1 entry by activating WIV1 S-mediated membrane fusion and partially bypassed IFTIM3-mediated restriction. (A), (B) and (C) Enhancement of TMPRSS2 on cell entry mediated by glycoproteins from Tor2, WIV1 and VSV, respectively. Tor2pp, WIV1pp and VSV-Gpp were used to infect T-Rex 293-ACE2 cells or T-Rex 293-ACE2-TMPRSS2 cells. At 48 h post-infection, luciferase activities were measured. Error bars reveal the standard deviation of the means from four repeats. (D) Syncytia formation between cells expressing S protein of Tor2 or WIV1 or VSV-G and cells expressing human ACE2 in the presence or absence of TMPRSS2. 293 T cells transfected with plasmids containing S gene were mixed with 293 T cells transfected with plasmids expressing human ACE2 and pCAGGS vector or TMPRSS2 plasmid at a 1:1 ratio. Twenty-four hours later, syncytia formation was observed. (E) ACE2-transfected FLIP-IN T-Rex 293-IFITM3 cells were cultured in the presence or absence of tetracycline (1μg/ml) for 24 h, then transduced with Tor2pp or WIV1pp. The transduced cells were treated with 5 μg/ml TPCK-trypsin or DMSO at 37°C for 13 min. Luciferase activities were measured at 48hpi. Relative infection refers to the ratio of the luciferase activity in T Rex293 cells cultured with Tet over that in cells cultured without Tet. Error bars reveal the standard deviation (n = 4). (F) T- Rex 293-ACE2 cells and T-Rex 293-ACE2-TMPRSS2 cells were cultured in the presence or absence of tetracycline (1 μg/ml) for 24 h, then transduce with Tor2pp and WIV1pp. Luciferase activities were measured at 48hpi. Relative infection refers to the ratio of the luciferase activity in T Rex293 cells cultured with Tet over that in cells cultured without Tet. Error bars reveal the standard deviation (n = 4).

Proteolytic cleavage of coronaviral S proteins is a critical priming step for membrane fusion [19]. Previous studies demonstrated that SARS-CoV utilizes cell surface transmembrane protease TMPRSS2 and airway trypsin-like protease to facilitate viral entry by activating spike-mediated membrane fusion at or near the plasma membrane of target cells [34,35]. Considering the high sequence identity between the S proteins of SARS-CoV and WIV1, we hypothesize that WIV1 can also exploit TMPRSS2 to cleave its S protein for cellular entry. To test this hypothesis, we expressed the TMPRSS2 in target cells and examined its effect on both viral entry and membrane fusion. As shown in Figure 5(A to C), the expression of TMPRSS2 significantly enhanced the infection by both Tor2pp and WIVpp, but not by VSV-Gpp, indicating that TMPRSS2 can specifically promote entry of SL-CoVs. Consistently, the syncytia formation induced triggered by the S protein and ACE2 interaction was also enhanced by TMPRSS2 expression (Figure 5(D)). To determine if the entry enhancement of TMPRSS2 can offset the restriction by IFITMs, we expressed TMPRSS2 or trypsin in the IFITM3 stable cells and tested the virus entry. The results showed that co-expression of trypsin almost completely rescued the entry of Tor2pp and WIV1pp from IFITM3 restriction (Figure 5(E)), while expression of TMPRSS2 rescued the entry of Tor2pp and WIV1pp by ∼70% and ∼60%, respectively (Figure 5(F)). These results indicate that WIV1 can also exploit TMPRSS2 to promote virus entry by activating membrane fusion and offset the inhibitory effect mediated by IFITM3.

### Amphotericin B treatment dramatically enhances the entry of SARS-CoV and SL-CoVss and compromises IFITM3-mediated restriction

**Figure 6.**
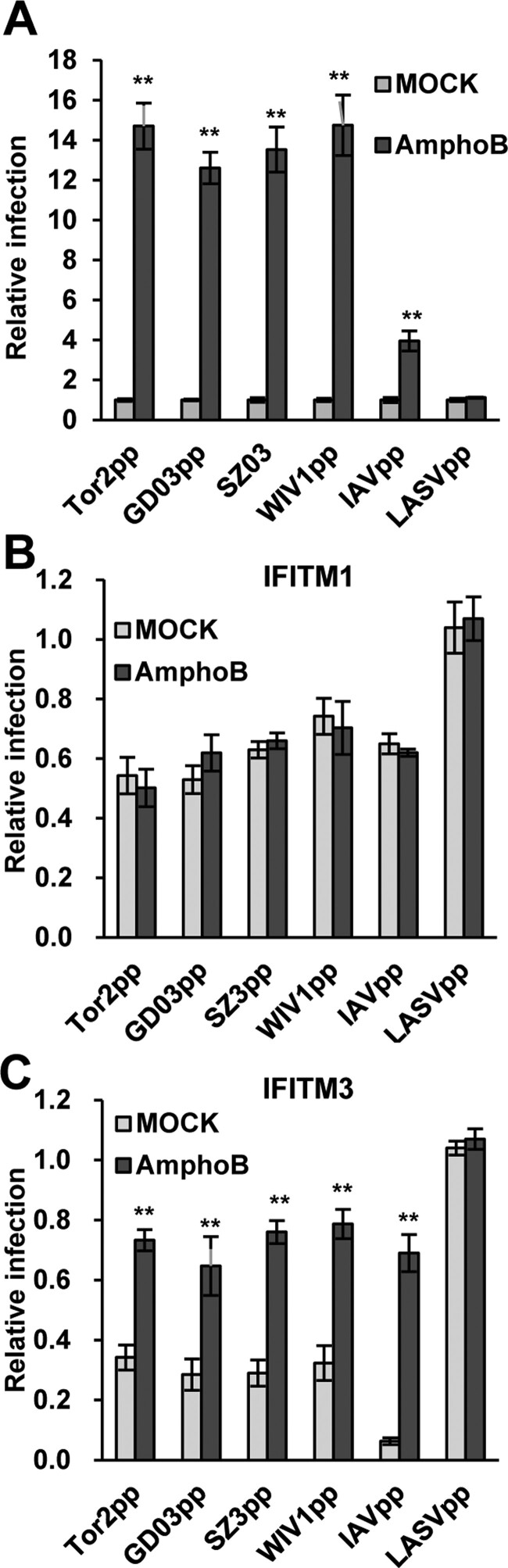
Amphotericin B treatment enhances the entry of SL-CoVs and SARS-CoVs and abrogates IFITM3-mediated restriction. (A) T Rex 293 cells transfected with ACE2 were infected with Tor2pp, GD03pp, SZ3pp, WIV1pp, IAVpp or LASVpp in the presence or absence of 1μM AmphoB. Luciferase efficiency was measured at 48 hr post-infection, and was normalized to DMSO-treated cells. The error bars refer to standard deviations (n = 4). (B) and (C) T-Rex 293 cells stably expressing IFITM1 or IFITM3 proteins were cultured in the presence or absence of Tet, and then were treated with 1μM AmphoB or DMSO. Twenty-four hours later, the cells above-mentioned were infected with Tor2pp, GD03pp, SZ3pp, WIV1pp, IAVpp or LASVpp. Luciferase efficiency was measured at 48 hr post-infection and normalized to cells cultured in the absence of Tet. The error bars refer to standard deviations (n = 4).

To investigate whether other agents can enhance SL-CoVs entry and evade IFITMs-mediated restriction, we tested an antibiotic, named amphotericin B (AmphoB), which is commonly used in clinics to treat systemic fungal infections and has been shown to neutralizing IFITM3-mediated restriction of IAV infection [54]. As illustrated in Figure 6(A), AmphoB treatment did not affect the infection of LASVpp, but enhanced the infection of IAVpp by ∼4 folds. Interestingly, AmphoB treatment enhanced the infection of lentiviral particles pseudotyped by the S protein of SARS-CoVs (Tor2 and GD03) or SL-CoVs (WIV1 and SZ03) by 13–15 folds. Moreover, AmphoB treatment compromised the restriction of SARS-CoVs and SL-CoVs by IFITM3, but not IFITM1 (Figure 6(B,C)). As a matter of fact, in the presence of IFITM3, the entry of SARS-CoVs and SL-CoVs still increased by ∼2 folds when receiving AmphoB treatment (Figure 6(C)). Nevertheless, these results imply that the patients receiving AmphoB treatment are at a higher risk to infection by SARS-CoVs and SL-CoVs.

## Discussion

In this study, we examined the potential host range of bat SL-CoV WIV1 and investigated the mechanism by which the virus evades the restriction of cell entry by host innate antiviral effectors. Results obtained from this study have important implications in the risk of cross-species transmission of bat SL-CoVs to human population.

Our results showed that bat SL-CoV WIV1 could use ACE2 orthologs from a broader range of animals as entry receptor than human SARS-CoV isolate Tor2 (Figures 1(C) and 2). Among those ACE2 orthologs tested, hACE2 exhibited the highest receptor activity while the others exhibited similar activities to support WIV1 S-mediated entry (Figure 1(C)). Consistent with previous studies [22–28], ACE2 orthologs of civet cat, ferret badger, hog badger, raccoon dog, and feline, but not rat and bat-Rs, were found to support cell entry of Tor2pp at a level of 50%–68% relative to that of hACE2 (Figure 1(C)). For WIV1, however, all the wild animal ACE2 orthologs tested here, including rat and bat-Rs, could be used as receptor at a level of 40%–88% relative to hACE2 (Figure 1(C)). Although these data were obtained by using HIV1-based pseudotyped virus system, for SARS-CoV, they were consistent with data obtained by using live virus [12,29–31]. For WIV1, virus infection data has shown that the virus titre at 48 h post-infection of HeLa cells expressing civet cat ACE2 or bat-Rs ACE2 was approximately two thirds of hACE2 [9]. These authentic virus infection data were amazingly consistent with our pseudotyped virus infection data (Figure 1(C)). Therefore, it is reasonable to postulate that WIV1 pseudotyped virus entry data for other ACE2 orthologs also reflects the live virus infection, meaning that WIV1 could cause infection in all the animals whose ACE2 were tested here. In particular, given that hACE2 had the highest receptor activity (Figure 1(C)), WIV1 may be more efficient to infect humans than its natural host bat-Rs. These findings underscore the possibility that WIV1 may directly jump to human population or use some wild animals as intermediate hosts and then jump to humans [55].

Although hACE2 exhibited the highest receptor activity among all the ACE2 orthologs tested here, we noticed that WIV1 has not fully evolved to use hACE2 as an efficient receptor. When compared to SARS-CoV Tor2, hACE2 receptor activity for WIV1 was only about 55%, a level similar to hACE2 usage by the mild SARS-CoV isolate GD03 and two civet cat SL-CoVs, B039G and A022G (Figure 3(B)), suggesting that even if WIV1 infects humans, it may only cause mild symptoms. Previous study in BALB/c mice model or hACE2-expression mice model has also shown that virus titre and/or symptoms were significantly attenuated for WIV1 compared with another severe SARS-CoV isolate Urbani [56]. We also found that changing N487 to T487 in the RBD of WIV1 did not enhance the binding to hACE2 and virus entry (Figure 3(B–D)). This is in sharp contrast to the observation in civet cat isolate SZ03 where changing S487 to T487 significantly enhance the hACE2 binding and virus entry [48]. Among 14 critical residues in the RBD of WIV1, there are two other residues (Y442 and L472) that are different between WIV1 and SARS-CoV Tor2. However, structural modelling predicted that all these three amino acid changes should not ablate receptor binding [56]. Therefore, in order for WIV1 to fully adapt to humans to cause severe symptoms, additional adaptation is required in the S gene and possibly also in other genomic regions.

Although receptor recognition is the major determinant of cell entry, host range, tissue tropism and pathogenesis [20], other host factors also contribute to the cell entry process by either restriction or promotion. In this study, we showed that, like SARS-CoVs, cell entry of WIV1 and three civet cat SL-CoVs were also strongly restricted by IFITMs (Figure 4). IFITMs are a group of interferon-induced transmembrane proteins that have been known to inhibit cell entry of a broad spectrum of viruses, including SARS-CoV and other human coronaviruses [43,45,57]. Interestingly, although these SARS-CoVs and SL-CoVs had different levels of RBD-hACE2 binding affinity and cell entry (Figure 3(B,C)), their susceptibility to IFITMs restriction was found to be at a similar level (Figure 4(B)). This suggests that IFITM restriction does not occur at the receptor-binding step during the entry of SARS-CoVs and SL-CoVs. This is consistent with previous findings that IFITM restricts SARS-CoV infection at a late stage in the endocytic pathway, most likely by inhibiting viral envelope protein-induced membrane hemifusion [43,58]. However, our finding is different from what was found in HIV-1 and Simian-Human Immunodeficiency Virus (SHIV), where IFITM limited the viral entry in an envelope glycoprotein-dependent manner [59–61]. While the V3 loop sequence of HIV-1 Env determined viral susceptibility to IFITM3 restriction of entry, IFITM3 impeded the cellular entry of all the tested SL-CoVs with similar efficacy [61]. Of note, we found that WIV1 and SARS-CoV could exploit TMPRSS2, a cellular serine protease constitutively expressed in airway, to promote entry by activating spike-mediate membrane fusion and partially offset the restriction by IFITM3 (Figure 5). TMPRSS2 has been previously shown to promote the entry of human CoV 229E and partially bypass IFITM restriction [46]. However, not every enveloped virus has successfully evolved to acquire this strategy. For example, the entry mediated by VSV-G protein was not enhanced by TMPRSS2 expression (Figure 5(C)). Whether or not WIV1 can exploit other cellular proteases, e.g. the endosomal protease cathepsin L or B, to promote its entry and overcome IFITM restriction is worthy of further investigation.

Interestingly, we found that AmphoB, an anti-mycotic drug commonly used for systemic fungal infections [62,63], could dramatically enhanced the cell entry of SARS-CoVs and SL-CoVs (Figure 6(A)), and completely blocked IFITM3-mediated restriction, but did not affect IFITM1-mediated restriction (Figure 6(C)). As a matter of fact, in the presence of IFITM3, the entry of SARS-CoVs and SL-CoVs still increased by ∼2 folds by AmphoB treatment. Previous study showed that mice treated with AmphoB developed more severe symptom upon infection by mild IAV isolate, similar to animals deficient in IFITM3 [54]. Therefore, those patients receiving AmphoB treatment may be at a significantly higher risk to infection by SARS-CoVs and SL-CoVs. This is particularly relevant at this moment as the newly emerging human CoVs, SARS-CoV-2, which shares high sequence identities and ACE2 receptor usage with the CoVs tested in this study [16,17], is still circulating globally. Although without direct examination, we strongly suggest that it should be very cautious to use amphotericn B for treatment of to COVID-19 with systemic fungal co-infection.

Our study has some limitations. First, due to the limitation by using pseudotyped particles but not live virus in this study, we have to made a careful conclusion that TMPRSS2 could enhance the WIV1 entry rather than infection or replication. Second, we examined the binding of ACE2 to RBD of SL-CoV by using immunoprecipitation, a semi-quantified strategy. More accurate method such as surface plasmon resonance using Biacore should be applied.

In summary, we found that bat SL-CoV WIV1 exhibits a broader range of ACE2 receptor usage than human SARS-CoV. Although hACE2 has the highest receptor activity for WIV1, the virus has not yet fully evolved to use hACE2 as efficient as SARS-CoV. Cell entry of WIV1 could be counteracted by host restriction factor IFITMs. However, WIV1 could exploit the airway protease TMPRSS2 to promote it entry by activating S-mediated membrane fusion and partially evade the IFITM-mediated restriction. Clinically, AmphoB treatment might dramatically enhance the infection by SARS-CoVs and SL-CoVs, and overcome the IFITM3-mediated restriction. These results further underscore the risk of exposure to animal SL-CoVs and highlight the vulnerability of patients who take AmphoB treatment to infection by SL-CoVs, including the most recently emerging SARS-CoV-2.
